# Blockchain based medical image encryption using Arnold’s cat map in a cloud environment

**DOI:** 10.1038/s41598-024-56364-z

**Published:** 2024-03-07

**Authors:** Saba Inam, Shamsa Kanwal, Rabia Firdous, Fahima Hajjej

**Affiliations:** 1 Department of Mathematical Sciences, Fatima Jinnah Women University, The Mall, Rawalpindi, Pakistan; 2 Department of Information Systems, College of Computer and Information Sciences, Princess Nourah Bint Abdulrahman University, P.O. Box 84428, 11671 Riyadh, Saudi Arabia

**Keywords:** Blockchain, Cloud computing, Arnold’s cat map, Orthogonal matrix, Structural similarity index measure (SSIM), Engineering, Mathematics and computing

## Abstract

Improved software for processing medical images has inspired tremendous interest in modern medicine in recent years. Modern healthcare equipment generates huge amounts of data, such as scanned medical images and computerized patient information, which must be secured for future use. Diversity in the healthcare industry, namely in the form of medical data, is one of the largest challenges for researchers. Cloud environment and the Block chain technology have both demonstrated their own use. The purpose of this study is to combine both technologies for safe and secure transaction. Storing or sending medical data through public clouds exposes information into potential eavesdropping, data breaches and unauthorized access. Encrypting data before transmission is crucial to mitigate these security risks. As a result, a Blockchain based Chaotic Arnold’s cat map Encryption Scheme (BCAES) is proposed in this paper. The BCAES first encrypts the image using Arnold’s cat map encryption scheme and then sends the encrypted image into Cloud Server and stores the signed document of plain image into blockchain. As blockchain is often considered more secure due to its distributed nature and consensus mechanism, data receiver will ensure data integrity and authenticity of image after decryption using signed document stored into the blockchain. Various analysis techniques have been used to examine the proposed scheme. The results of analysis like key sensitivity analysis, key space analysis, Information Entropy, histogram correlation of adjacent pixels, Number of Pixel Change Rate, Peak Signal Noise Ratio, Unified Average Changing Intensity, and similarity analysis like Mean Square Error, and Structural Similarity Index Measure illustrated that our proposed scheme is an efficient encryption scheme as compared to some recent literature. Our current achievements surpass all previous endeavors, setting a new standard of excellence.

## Introduction

The Internet has benefited humanity in numerous ways, the most notable of which is by providing a platform for virtual environments. Today, every industry is aggressively attempting to virtualize its operations using web applications. While the Internet and web apps have a positive reputation, they also have a negative reputation for being a growing hive for criminal activities such as online identity theft and fraudulent transactions. As a result, researchers have taken a keen interest in making the Internet and online applications more secure and trustworthy^1^. For this, a new technology known as “cloud computing” has been introduced.

The concept of network-based computing dates to the early 1960s, although many believe the term “cloud computing” was first employed in its contemporary meaning on August 9, 2006, when Google CEO Eric Schmidt introduced it at an industry conference^2,3^. Cloud computing has grown significantly in industrial technology such as Amazon, IBM, HP, Apple, and Oracle^4–6^. It is widely used in the telemedicine industry as well. Telemedicine is a fast-growing field in which medical care is provided to patients who aren’t physically available at the same place as the physician^7^.

Furthermore, safe internet exchanges of patient medical information, including medical scans, are becoming more widespread. Because medicinal data is usually available in a visual manner, it must be strictly protected. Therefore, a complicated medical system requires expertise capable of accumulating medical documents in a format that allows authorized users to access them from any location^8,9^. As we all know, medical data is growing by the day, and we need to preserve it for future use. To properly handle such a complex and vast amount of data significantly, specific technologies such as a distributed data network, scalable storage, parallel processing, frameworks, infrastructures, and so on are necessary. Because cloud computing is based on service-oriented architecture, it can handle these complex problems in a virtualized environment at a minimal cost^10,11^.

Furthermore, cloud computing security issues arise because of individuality and risk management, integrity control, public services, and data access management. Individual e-mails and contact numbers can be protected from being accessed by attackers who wants to get information by validating the sender’s identity^12^. Several solutions have been used to overcome the cloud communication problem in terms of privacy and security, but there are some restrictions. Keeping sensitive data on cloud computing is a major concern that necessitates including the defense problem that occurs on cloud computing. At this time, conventional system has been enormously enhanced to offer safety measures in the health care industry, but they include elevated prices and greater usage of computation resources in the meantime^13^.

As a result, preserving the medical images whilst retaining its reliability is critical. Data integrity refers to the assurance that the medical images doesn’t change by not permitted parties. An illicit operator has the capacity to change medical images. As an outcome of the data modification or erosion in the picture, disease diagnosis will be incorrect. As a result, a strong and dependable technique for safely sending delicate health care over public channels is required^14^.

For this, before sending data to a cloud server, we must first encrypt it so that no one, not even the cloud server, can read it. Traditional cryptosystems, like AES^15^ and DES^16^, are unsuitable for quick image encryption because they need a large amount of computational power and a long time to complete. Many encryption algorithms have been developed to meet the criteria of security, privacy, and efficient calculation. Chaotic theory-based encryption approaches are ideal for image encryption because they provide high-level of security, greater speed, processing capacity, and process complexity. Non-periodicity, sensitivity to beginning circumstances, and unpredictability are all characteristics of chaotic maps. These are employed in picture encryption for data confusion and dispersion. The privacy and security of information are enhanced by chaotic maps. That’s why we use two different chaotic maps in our proposed scheme i.e., Henon map and Arnold’s cat map (ACM) for making the encryption scheme key sensitive orthogonal matrix generated from an equation of plane along with hill cipher is used.

Sending encrypted data using chaotic mappings over public channels is secure but we need more security as well as data authentication and data integrity so that the receiver at the other end will get assured that decrypted image and the original image is same. To address these concerns, the use of blockchain is proposed in this model. Blockchain technology has captured the attention of many stakeholders, including healthcare, finance, real estate, infrastructure, and governmental organizations^17,18^.

Using blockchain to store and manage electronic health records will be efficient and safe. The combination of blockchain and cloud storage to process healthcare data will deliver high-quality services at a reasonable cost^10^. For its services, the blockchain network does not rely on a centralized, trustworthy third party. It is a decentralized network in which there is no single point of failure. If one or more nodes fail, there is enough redundancy to keep the rest of the network operational^19^.

### Contribution

The previous frameworks include image encryption in cloud environment or transaction of data through blockchain. This study merges both concepts as we want a scheme where we can share sensitive data like medical images all over the world along with the confidentiality of the data. For this purpose, our proposed scheme contributes in the following ways:

- We adopt the same model as given in ref^20^. To enhance security, this model is modified by our new proposed encryption scheme. This new proposed encryption scheme exhibits better results and performance. Different results and analysis are shown in Table 6a.
- The chaotic based Arnold’s cat map with orthogonal key matrix encryption scheme uses three different keys with large key space, complexity, randomness, and high complexity. It keeps the intruders from gaining access to original medical images.
- The BCAES protects personal medical information and validates medical data using blockchain technology. As a result, privacy and data integrity have both improved.
- To check the integrity and authenticity of medical images, the concept of blockchain along with cloud server is introduces where the signed document of images is stored in blockchain, and data user can easily validate the image after verifying it through the blockchain.

The remainder of the paper is structured as follows: Section “Related work” illustrates related works of the scheme. Section “Mathematical preliminaries” is about mathematical preliminaries. Section “Proposed blockchain based chaotic Arnold’s cat map encryption scheme (BCAES)” presents the proposed BCAES and the encryption/decryption process of the scheme. Section “Comparative results and performance evaluation” is devoted to performance evaluation. Conclusion and future work will be discussed in Section “Conclusion”.

## Related work

Mondal and Goswami^21^ presented an operative honeypot technique for cloud computing documents protection. Initially, a normalization procedure substitutes for and eliminates undesirable missing values from the collection. Following that, the GLCM quality selection algorithm and CNN classifier predict and categorize the assault types^22^. In this strategy, a cryptographic technique (honeypot) is used for encrypting the data. The CS is responsible for the key creation and key verification as well with the user for authentication. After that, the data owner requests information from the cloud server. Honeypot technique is used to decrypt the information after entering the key by the user. This gives valuable security, but at a great expense.

Ali et al.^23^ demonstrated an encrypting technique for health care data built on a Henon chaotic map and a logistic tent map. In this encryption approach, the user produces a private encryption key by utilizing the medical center’s public key. The proposed chaos-based medical image encryption method then encrypts with a private key. After that, the user digitally signs the encrypted image and sends it along with the digital signature and authentication parameters to the administrators. Finally, using a cipher image, secret key, and a digital signature, the healthcare corporation constructs the shared secret key. The authority then uses that secret key to decrypt the picture, checks it using an authenticating factor, and validates it if it is authorized.

Padhy et al.^24^ proposes a high-level, cloud-based rural health records system that delivers low-cost services to rural residents. They have saved personal details in a cloud-based system that authorized medical researchers and doctors may use for better medical facilities and illness diagnostics in remote places. Patients have privileged access to their health information and medicines at any time for prompt treatment. This technique is less expensive than others and removes the time and other procedures required to register at a hospital.

Neela and Kavitha^20^ proposes a Blockchain based, secured model known as BCDGE. In this model, the health care data is kept in the cloud after applying the encryption technique using Chaotic Deep GAN. It generates the private key, which is then followed by the processes of confusion and diffusion. Then the XOR approach decrypts the image by using private key that was generated. Then the sender uploads the cipher image into the cloud, makes a digital signature of it using the hash function, and stored it into blockchain. Then the digital signature may be utilized to validate the ciphertext image’s validity. The suggested Chaotic Deep GAN approach features pseudo randomness, a large key space, and is particularly sensitive to alteration, according to experimental results and security studies.

For MI cloud storage, Lakshmi et al.^25^ presented the HNN-IES (Hopfield Neural Network Images Encryption Scheme). This structure is divided into five phases. The underlying stage depicts flexible key generation using a recursive neural network. Following that, the stage shows a photograph of an explicit and unpredictable arrangement generated using HNN. The confusion and diffusion measures are therefore arranged separately in phases 3 and 4. Finally, it demonstrates the operational connection between the cloud and cryptosystem.

MeDShare^26^ is a cloud-based healthcare data sharing platform that uses blockchain technology. The authors of this system employed smart contracts and authentication and authorization on data access via a separate platform. When transferring healthcare data between cloud structures, MeDShare can safely accomplish and maintain data authenticity and accountability. ProvChain^27^ is a cloud-based, strong authentication system designed to improve availability and address privacy concerns. This technique is completely decentralized and relies on cloud computing for tamper-proof access through blockchain technology.

To protect medical images in the cloud, Afzal et al.^28^ developed the biplane and Chaotic Image encryption. Initially, a biplane and chaotic encryption (BCE) used for encrypting the medical images. The chaotic key sequence key is obtained by merging the two keys obtained using the logistic map and a linear feedback shift register. Finally, after effectively retrieving the cipher image from the CS, the technique is employed to extract the plain medical image. This approach has a significant computational cost.

## Mathematical preliminaries

The following mathematical ideas are used in our proposed encryption scheme: The Henon map, the orthogonal matrix, and the Arnold’s cat map (ACM). Chaotic maps are the basic maps that are affected by their initial parameters. A small modification in the initial conditions can have a huge influence on the outcomes.

### Henon map

Michel Henon created the Henon map in 1969. It is a descrete dynamic map wit chaotic behavior due to its sensitivity to initial parameters. Mathematically, it can be expressed as follows:

1 $$\left. {\begin{array}{*{20}l} {Y_{n + 1} = 1 - a Y_{n}^{2} + b Z_{n} } \hfill \\ {Z_{n + 1} = b Y_{n} } \hfill \\ \end{array} } \right\}$$

The violent behavior of chaotic system is determined by the values of the control parameters $a$ and $b$. The Henon map’s parameters and conditions are as follows:

1. ${Y}_{0}$, where ${Y}_{0}$ is the initial value.
2. $a$ is the controlled parameter, where $a\epsilon \left[\mathrm{0,1}\right[$.
3. ${K}_{1}$ is the secret key for the permutation phase in encryption, where ${K}_{1}=\left(a,{Y}_{0}\right)$

For $a=1.4, {Y}_{0}=0.631, b=0.3, {Z}_{0}=0.189$, this structure is chaotic. Eventually, a slight modification in parameter values might cause a system to behave differently.

It has various beneficial characteristics, including the Lyapunov exponent, unpredictability of behavior, and uniform non-variation of the intensity variable. Because of such qualities, the Henon map is highly suggested for cryptographic functions.

### Orthogonal matrix

A matrix T is considered to be orthogonal if and only if it has the following properties:

2 $${T}^{t}T=I$$

3 $${T}^{t}={T}^{-1}$$

where ${T}^{t}$ is called the transpose of T where $I$ is the identity matrix. During encryption this orthogonal matrix T is calculated using a plane equation $ax+by+cz=d$, where $a,b,c,d\epsilon {\mathbb{R}}.$

### Arnold’s cat map (ACM)

ACM is a well-studied example of a discrete system with chaotic behavior^29^. In 1960, V. Arnolds come across Arnold’s cat map (ACM). He incorporated a cat picture into his work. Assuming pixel image as $P=\left\{\left(x,y\right) \right| x,y=\mathrm{0,1},2\dots .N-1\}$, 2-D ACM can be written as:

4 $$\left[\begin{array}{c}x{\prime}\\ y{\prime}\end{array}\right]=A\left[\begin{array}{c}x\\ y\end{array}\right] \left(mod n\right)$$

5 $$A=\left[\begin{array}{cc}1& p\\ q& pq+1\end{array}\right] \left(mod n\right)$$

where $p,q$ are positive integers, such that $\left|A\right|=1$. It can also be written in the form of equation by taking $p=1, q=1$ as:

6 $$\left. {\begin{array}{*{20}l} {x_{n + 1} = 2x_{n} + y_{n} } \hfill \\ {y_{n + 1} = x_{n} + y_{n} } \hfill \\ \end{array} } \right\}$$

By using ACM, it generates an arbitrary image in the intruder’s eyes by shuffling all the image’s pixels. As a result of seeing the shuffled image, the attacker becomes confused and is unable to establish the accuracy of the image, which is used in the encryption process. So, the parameter R (number of iterations) in Arnold’s cat map technique can be used as a secret key ${K}_{3}$.

## Proposed blockchain based chaotic Arnold’s cat map encryption scheme (BCAES)

**Figure 1:**
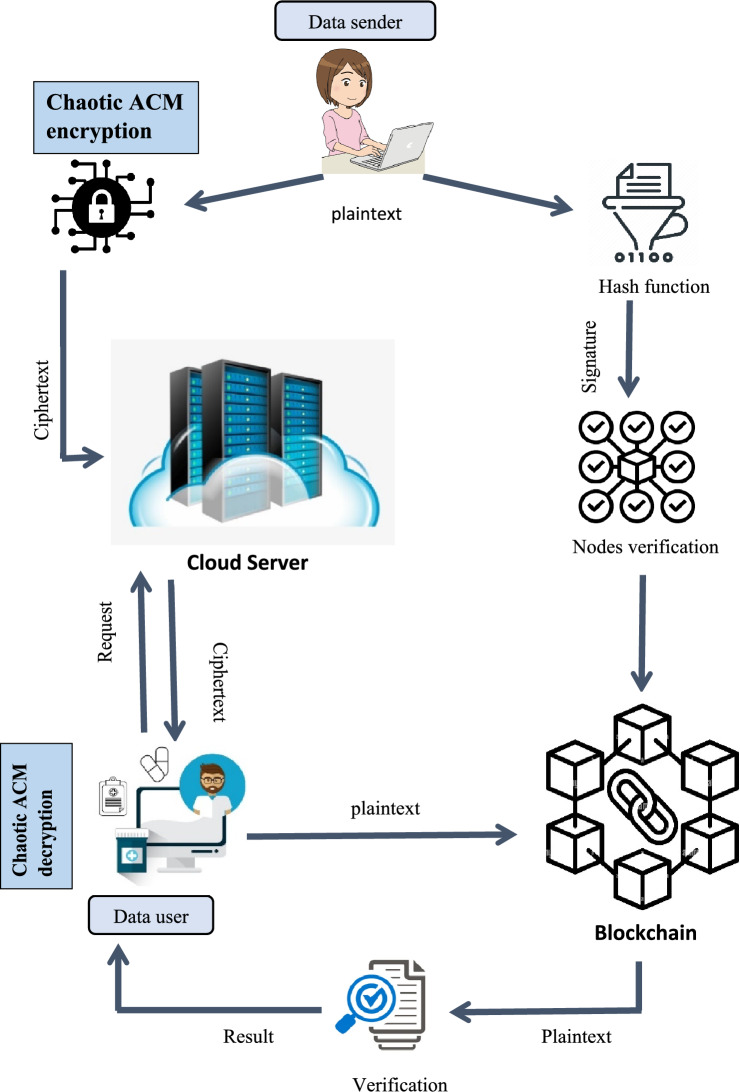
Model of proposed BCAES.

The five primary components of the BCAES are the data sender, CS, data user, blockchain, and encryption/decryption process. The system model of the BCAES is depicted in Fig. 1. Initially, the sender encrypts the medical images and makes a digital signature of it through the hash function SHA-256. Then the sender will encrypt the medical image using the Image encryption algorithm which is discussed in Section “Image encryption algorithm” and then encrypted image will be stored in cloud server and the signed document will be stored in blockchain. When the data user wants the medical image, he will place a request for ciphertext to cloud server. Then, the cloud server will send the relevant encrypted file to the user. After getting the cipher image, the data user will decrypt it using ACM decryption process illustrated in Algorithm 5. Now to check the integrity and authenticity of image, data user will send the decrypted file into blockchain and blockchain will validate it and sends a verification message in the form of yes or no. The primary components of our proposed model are listed below:

- *Data Sender* Data sender (who might be patients) encrypts the medical images using the ACM encryption scheme and sends the retrieved medical information to CSP. The data sender also signed the encrypted image, which is then saved into the blockchain network.
- *Cloud Server (CS)* The cloud server has two goals:
  - To store massive amounts of medical image data
  - The other is to seek for and sends the correct ciphertext in response to the data user’s request.
- *Data user* To obtain the encrypted image, the data user (health professionals) requests the health care information from the CS. Furthermore, the users validate the ciphertext’s validity by cross-checking the ciphertext’s ID saved in the blockchain.
- *Blockchain* For creating digital signature, we used the SHA-1 algorithm to create a hash value of the image. When the data user requests a validity check, the blockchain validates the stored signature to authenticate the ciphertext’s authenticity. If it is true, it returns 1; otherwise, it returns 0.
- *Encryption/Decryption* The medical image is first encrypted using Arnold’s cat map (ACM) with an orthogonal key matrix and Henon map before being sent to the cloud server, whereas the inverse process will be the decryption process.

### Image encryption algorithm

**Figure 2:**
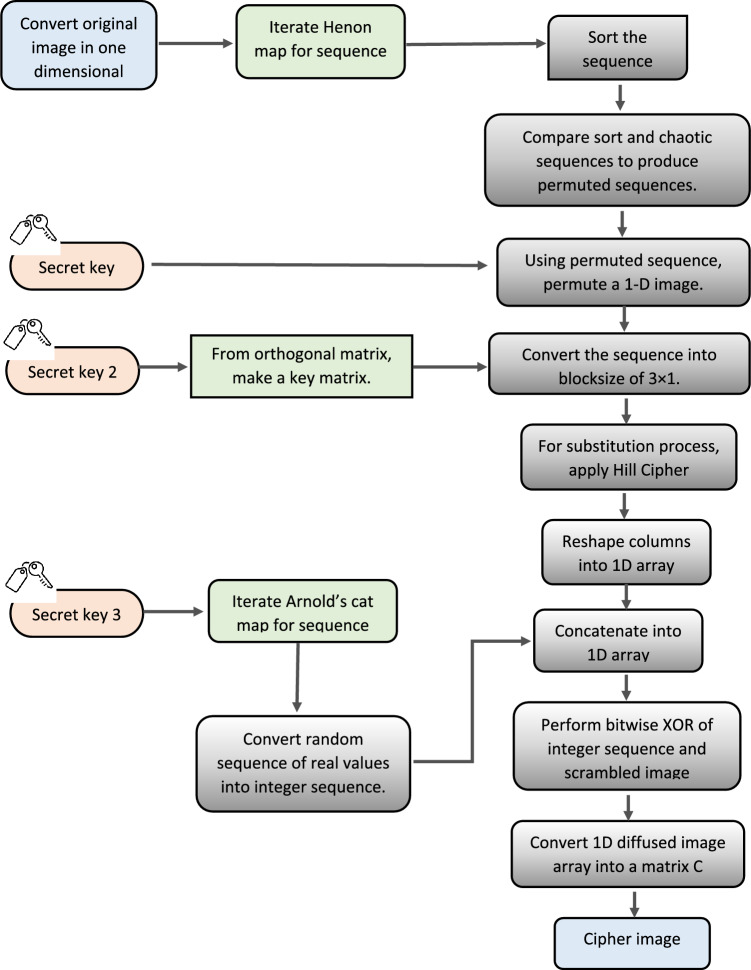
Workflow of encryption algorithm.

The image encryption algorithm is split into three phases. The first phase (confusion) uses Henon map to construct a sequence for permuting image pixels. The permuted pixels are combined with the key invertible matrix formed by a secret orthogonal matrix in the second step (permutation)^30^. The last phase (diffusion) consists of a unique sequence created from a new Arnold’s cat map that is XORed with earlier obtained outcomes. The density of the proposed scheme contributes to its resistance to attacker attempts. The process of our proposed encryption algorithm is shown in Fig. 2.

#### Permutation phase

As demonstrated in Algorithm 1, the permutation phase of our proposed encryption scheme comprises permuting the location of the pixels in an original image. In the first phase of our strategy, we use the Henon map with key ${K}_{1}$ to permute the pixel locations^31^. The Henon map is repeated using ${K}_{1}$ to generate a sequence. The chaotic sequence that is generated is sorted in ascending order. By comparing the structures of chaotic and sorted sequences, the permuted sequence is obtained. Using the permuted sequence, the original image’s one-dimensional array is recovered. The typical rule for picture variety is to choose any volume of $P\times Q\times 3$ pixels colorful picture, where Q and P are the width and height, correspondingly. The size of the original image and the encrypted image will remain unchanged.

**Algorithm 1:**
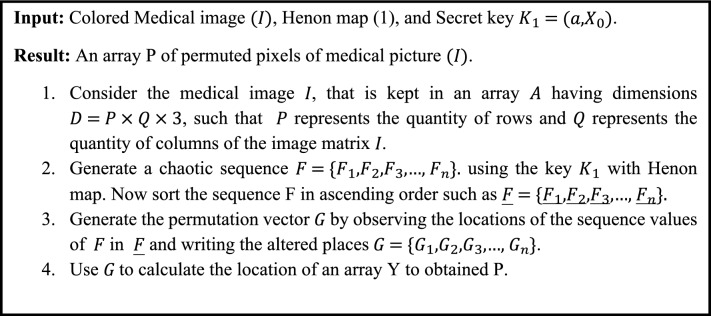
Pixel Permutation

#### Substitution phase

In this phase, the key ${K}_{2}$ is expressed as the orthogonal key matrix, which is created from an equation of a plane as shown in Algorithm 2. After generating key ${K}_{2}$, Hill cipher will be applied to get an array E of same size as D in permutation phase. To generate an array E, firstly the permuted picture is sub-divided into $D/3$ blocks. These $D/3$ sub-sections are then multiplying with ${K}_{2}$ separately. After that the result will be arranged in 1-D Array E. Mathematical implementation of substitution phase is discussed in Algorithm 3.

**Algorithm 2:**
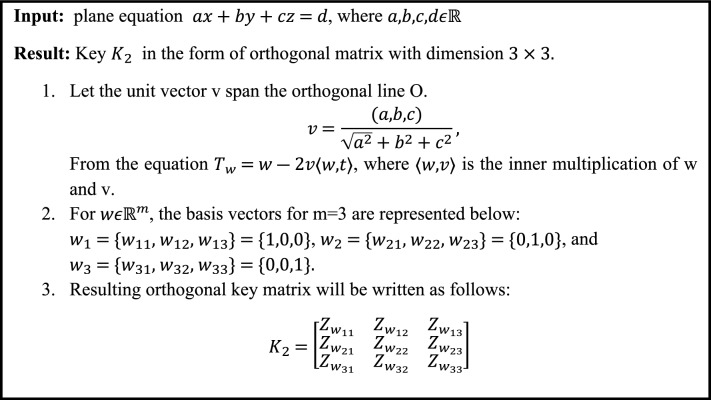
Key generation for substitution phase

**Algorithm 3:**
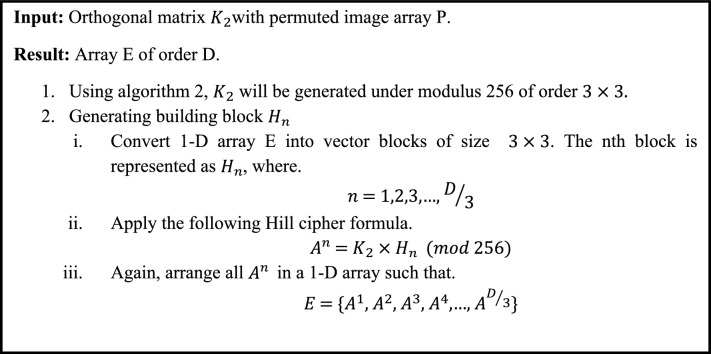
Applying Hill cipher with orthogonal key matrix.

#### Diffusion phase

In final phase of the encryption scheme, the diffusion of pixels is illustrated in algorithm 4. In the last phase, a key ${K}_{3}$ is used to generate a sequence using Arnold’s cat map (ACM)^28^. Using Eq. (7), the standards of the generated sequence are modified into an integer sequence. The 1-D Array E is then bitwise XORed with the corresponding integer sequence generated by ACM. A matrix for the encoded picture has similar size as original picture is generated by rearranging the 1-D array.

**Algorithm 4:**
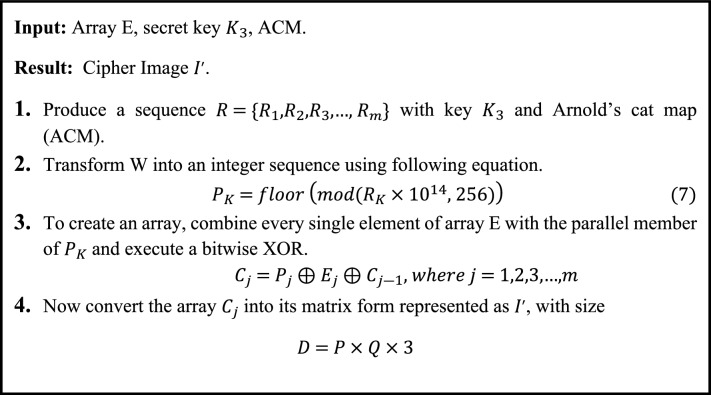
Diffusion of pixels

### Image decryption process

The reverse encryption method is used in the picture decryption process to get the original image. Algorithm 5 depicts three steps of the suggested decryption technique. In the first step, the Arnold’s cat map (ACM) sequence is XORed with the key ${K}_{3}$. ${K}_{2}$ is used to implement the Hill cypher using the invertible matrix. The Henon map is used to create a random sequence, and the inverse permutation is achieved by employing the key ${K}_{1}$. The inverse permutation is used to reverse the permutation. To acquire the original image, the preceding array is translated into an image form^32^.

**Algorithm 5:**
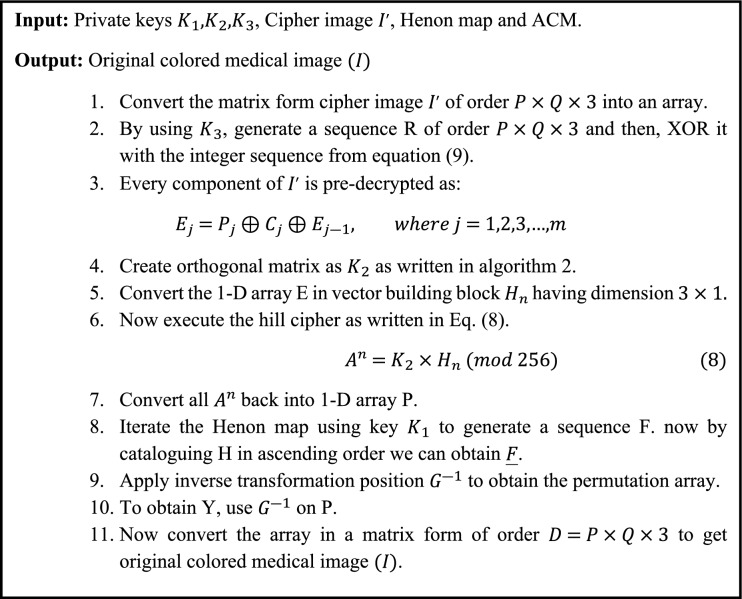
Decryption process

### Signature creation

Once the encryption procedure is complete, the sender transmits the secured health care data to the cloud server (CS) and uses a hashing algorithm to store the hash value of the medical image on the blockchain. This might be done for ciphertext integrity and authenticity. In our proposed scheme, we will apply SHA-256 to form a hash value (signature) of the encrypted image so that attackers cannot get access to the image as hash functions are one-way functions. It is impossible to decrypt the hashing value back to the original medical image^33^.

### Signature verification

**Figure 3:**
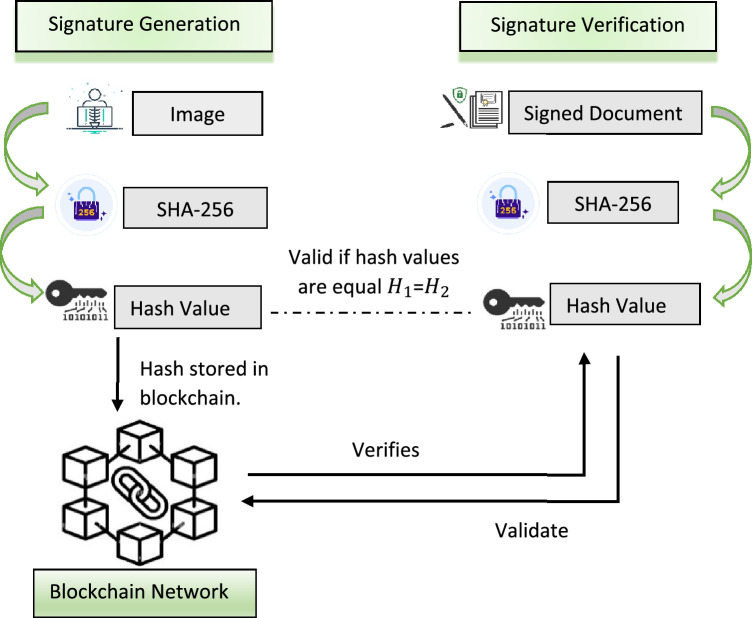
Model of signature generation and verification.

Finally, the user verifies the signature stored into the blockchain to ensure the validity of the ciphertext. When the data user sends the decrypted image into the blockchain. The blockchain will create a hash value of it using SHA-256 and then matches the hash value of decrypted image with the hash value of original image stored into the blockchain by data sender. If it matches up, the data is authentic; otherwise, it is not. If the data is authentic or not, the blockchain will sends a verification message in the form of yes or no to data user. The signature verification method is depicted in Fig. 3.

## Comparative results and performance evaluation

The tests were accomplished on a UBUNTU 16.04 desktop computer equipped with an Intel(R) Core (TM) i7-6700 @ 3.40 GHz processor. The simulation is conducted using Matlab 2018a. To simulate the suggested system, we take a private blockchain using the Geth Ethereum client. Ethereum is a popular blockchain platform, and its performance has been studied by developers and researchers. Table 1 lists the software utilized for implementation.

**Table 1:** Setup of parameters.

Software	Use	Version
Windows	Operating system	8.1
Mist	Ethereum wallet	0.9.2
Geth	Command line Ethereum client	1.7.2
Claymore pool	Claymore’s Dual Ethereum AMD GPU Miner	10
Cloud	Average RAM	512 MB
	Number of virtual machines	34
	Number of users	100
	Average bandwidth	1,000,000 MB

The sample images are saved from BraTS18 dataset^34^, the Ultrasonic Brachial Plexus dataset^35^ and the Montgomery country chest X-rat dataset^36^, because they represent three different anatomical regions. The sample images are colored with pixel values of length $\left(256\times 256\right)$. After encrypting the images we get the encoded image of same size i.e. $\left(256\times 256\right)$. When using a decryption technique, the encoded image and the plain image are recovered employing the methods of pixel permutation by the Henon map, pixel substitution using hill cipher with orthogonal key matrix, and pixel dispersion by Arnold’s cat map. The result obtained from our proposed encryption scheme is shown in Table 2. In our proposed encryption scheme, we use ${K}_{1}=\left(\mathrm{0.631,0.189}\right)$, ${K}_{3}=\left(\mathrm{0.015,0.223}\right)$, and

**Table 2:** Encryption and decryption result of our proposed scheme.

Sr. No.	Original	Encrypted	Decrypted
1			
2			
3			
4			
5			

$${K}_{2}=\left[\begin{array}{ccc}0& 204& 153\\ 204& 113& 20\\ 153& 20& 144\end{array}\right]$$

### Security analysis

We will test our encryption scheme by some sort of security analysis mentioned below like key space analysis, key sensitivity analysis, Histogram analysis, Chi-square analysis, Information entropy etc.

#### Key space analysis

In essence, key space analysis examines every possible key that may be used during encryption. The key’s size must be sufficient to avoid brute force attacks. If the key space is greater than 10^30^, an algorithm can avoid exhaustive attacks using existing statistical methods^37^. Our proposed encryption scheme depends upon three different keys. Henon and Arnold’s cat map’s control parameters make up the keys ${K}_{1}$ and ${K}_{3}$, respectively. The overall number of chances to select the keys might be $({{10}^{15})}^{2}\times ({{10}^{15})}^{2}={\left(10\right)}^{60}\approx {\left(2\right)}^{240}.$ In our suggested encryption scheme, first and last phase is secure enough to be protected against a brute force attack, even if the size of the keys for two algorithms can be up to 60. Consequently, ${K}_{2}$’s key space is infinite in size, as there are unlimited alternatives for selecting the four coefficients a, b, c, and d since the second key ${K}_{2}$ for the substitution phase is produced using an equation of the plane $ax+by+cz=d,$ where $a, b, c, d\epsilon {\mathbb{R}}$.

#### Key sensitivity analysis

The scheme’s secret keys are fundamental to its encryption scheme. Three keys make up the encryption method we suggest. With the current approach, even a very slight modification to any portion of the secret key causes a complete change in the decryption algorithm’s result. This indicates that if we modify the first key ${K}_{1} = (a, {X}_{0})$ by adding 0.0000000000000001, we will not be able to retrieve the original medical image using that key. It’s obvious that the encrypted image lacks any hints or gestures from the original image. Our suggested cryptosystem’s algorithms are extremely vulnerable to secret keys.

#### Information entropy

**Figure 4:**
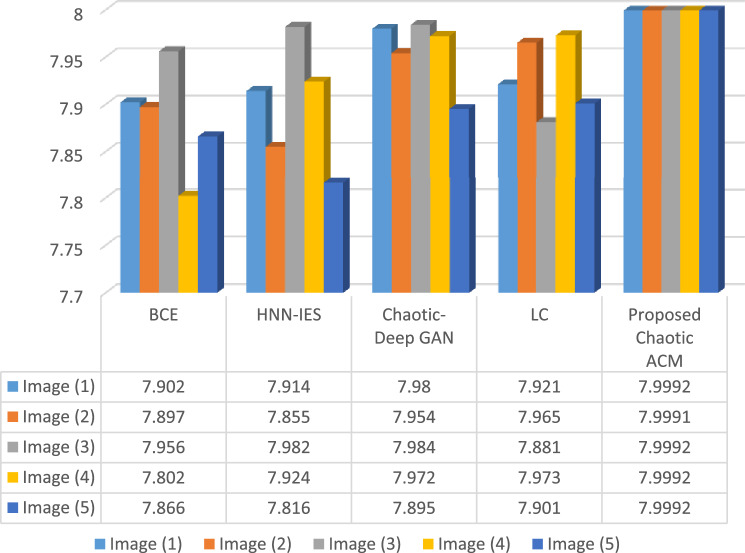
Comparison of entropy result for various methods.

This measurement has been used to assess the degree of uncertainty and quantify the randomness or instability of a private key. Equation (9) is used to determine the information entropy of images.

9 $$\mathop \sum \limits_{i = 0}^{m - 1} P\left( {m_{i} } \right)log_{2} \frac{1}{{P\left( {m_{i} } \right)}}$$

where *m* is the quantity of pixels, as well as $P\left({m}_{i}\right)$ stands for the chance that pixel $\left({m}_{i}\right)$ will appear^38^. The maximum entropy for images is 8. The acquired private key has a fair amount of unpredictability, as demonstrated by its entropy, which is around 7.9992. Comparing different encryption techniques with our proposed scheme, Fig. 4 shows the entropy result for the images shown in Table 2.

#### Chi-square test analysis

It can support the regularity in the histograms of the encrypted images. The excellent consistency in encoded image histograms is demonstrated by the low chi-square value. It can be evaluated using Eq. (10).

10 $${{Y}_{r}}^{2}={\sum }_{i-0}^{255}\frac{{\left({O}_{i}-{E}_{i}\right)}^{2}}{{E}_{i}}$$

where ${E}_{i}$ is the expected frequency and ${o}_{i}$ is the observed frequency of i. Using Eq. (11), ${E}_{i}$ (expected frequency) can be determined.

11 $${E}_{i}=\frac{size of an image}{26}$$

**Figure 5:**
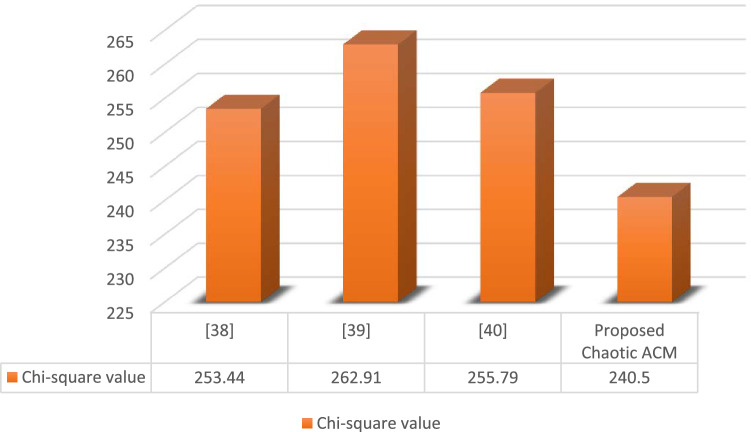
Chi-square test analysis.

Figure 5 compares the Chi-Square value of our proposed chaotic ACM with existing techniques. It is clearly demonstrated from the figure that our proposed encryption scheme has high level of consistency as compared to other existing techniques.

#### Histogram analysis

Histogram analysis is a revolutionary method of evaluating image pixels. It must be distinguishable from the encrypted and original image. The simple image’s pixels are constantly different and non-uniform. It is evident that the cypher image’s histogram is largely uniform. It is clear, that the dispersion of pixels in the original image’s cypher image does not provide any information^30^. The three parts of the histogram for the original and cypher images—red, green, and blue—are displayed in Table 3. By Table 3, the histogram of cipher images is reasonably uniform. Regarding the dissemination of pixels in the original medical image, there is no proof. Thus, it becomes very challenging for hackers to retrieve useful information from the encrypted images.

**Table 3:** Histogram analysis.

Plain and cipher images	Images	Histogram
Image 1		
Cipher image 1		
Image 2		
Cipher image 2		

#### Sensitivity analysis

The one-pixel value of the original picture is changed at random during the study. The suggested encryption method is then applied to the two images to generate two sets of private keys—one before and one after changing a pixel value. The variations between two private keys are now estimated using two methods to measure their sensitivity. Two metrics—the Number of Pixel Change Rate (NPCR) and the Unified Average Changing Intensity (UACI)—are used to assess the deviation between the secret keys. The experiment demonstrates how even minor changes to the plain image may have a major influence on the encrypted images. When a greater value of NPCR is attained, a more secure cryptosystem is built that will protect against a variety of attacks. Equations (12) and (13) shows the formulas to calculate the NPCR and UACI respectively.

12 $$NPCR=\frac{\sum_{i=0}^{m}\sum_{j=0}^{n}R\left(i,j\right)}{{I}_{s}}\times 100$$

13 $$UACI=\frac{1}{{I}_{s}}\left[\sum_{i=0}^{m}\sum_{j=0}^{n}\frac{\left|X\left(i.j\right)-X{\prime}(i,j)\right|}{256}\right]\times 100$$

And,

14 $$R\left(i,j\right)=\left\{\begin{array}{c}1, {R}_{1}(i,j)\ne {R}_{2}(i,j)\\ 0, {R}_{1}(i,j)={R}_{2}(i,j)\end{array}\right.$$

where ${R}_{1}$ and ${R}_{2}$ represent the pixel values at location $(i,j)$, and ${I}_{s}$ represents the size of the image.

**Figure 6:**
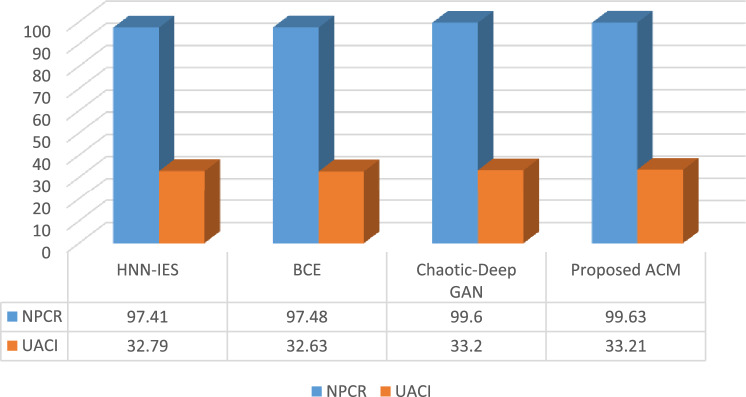
Comparison of NPCR and UACI valued for different methods.

According to Fig. 6, a little change in the actual image’s pixel value resulted in changes between generated private keys of over 99.63%, by average intensity changes exceeding 33%. It indicates that the generated secret key is sensitive to the original image and thus fulfills both randomness and uncertainty.

#### Correlation analysis of nearby pixels

The correlation coefficient demonstrates similarities between contiguous pixels in the diagonal, horizontal and vertical ways. The confusion and diffusion processes between the original and encrypted image are tested using correlation $Cr$. It may be computed using Eq. (15).

15 $$Cr=\frac{m(\sum_{i=1}^{n}{p}_{i}{q}_{i}-\sum_{i=1}^{n}{p}_{i}\sum_{i=1}^{n}{q}_{i})}{(m\sum_{i=1}^{n}{({p}_{i})}^{2}-{\left(\sum_{i=1}^{n}{p}_{i}\right)}^{2})(m\sum_{i=1}^{n}{{q}_{i})}^{2}-{\left(\sum_{i=1}^{n}{q}_{i}\right)}^{2})}$$

wherever, ${p}_{i}$ and ${q}_{i}$ are the values of two nearby pixels, and $m$ is the total pixel value used to calculate the coefficient. The maximum correlation factor value of 1 indicates that there is a strong association between neighboring pixels^39^. In order, to prevent an attacker from obtaining the necessary data, the proposed cryptosystem must employ low correlation coefficients that are close to zero. Table 4 displays the correlation distribution values for the original and cypher images in three different orientations. The pixels RGB component distribution of the encrypted medical images is shown in Table 5 vertically, horizontally, and diagonally. The data demonstrate that neighboring pixels in the encrypted image are not correlated since they are nearer to 0. In comparison, 16,430 pairs of random pixels are used, along with 4500 pairs of randomly selected surrounding pixels.

**Table 4:** Correlation coefficient values.

Direction/color		Horizontal	Vertical	Diagonal
Blue	Original	0.9776	0.9759	0.9568
	Encrypted	0.00007	0.0044	-0.0019
Green	Original	0.9668	0.9688	0.9402
	Encrypted	0.0036	-0.0006	-0.0030
Red	Original	0.9597	0.9711	0.9365
	Encrypted	0.0009	0.0064	-0.0027

**Table 5:** Correlation analysis of adjacent pixels for proposed cryptosystem.

Image 1	Cipher image 1
Correlation coefficient (horizontal wise) of cipher image 1	
Correlation coefficient (diagonal wise) of cipher image 1	
Correlation coefficient (vertical wise) of cipher image 1	

Image 2	Cipher image 2
Correlation coefficient (horizontal wise) of cipher image 2	
Correlation coefficient (diagonal wise) of cipher image 2	
Correlation coefficient (vertical wise) of cipher image 2	

### Similarity analysis

To preserve the standard of medical image in our proposed cryptosystem, we will apply some similarity analysis like Mean Square Analysis (MSE), Structural similarity Index Measure (SSIM), Peak Signal Noise Ratio (PSNR) etc. And then compare our proposed schemes with already existing techniques like BCDGE, HNN-IES and BCE.

#### Mean square analysis

**Figure 7:**
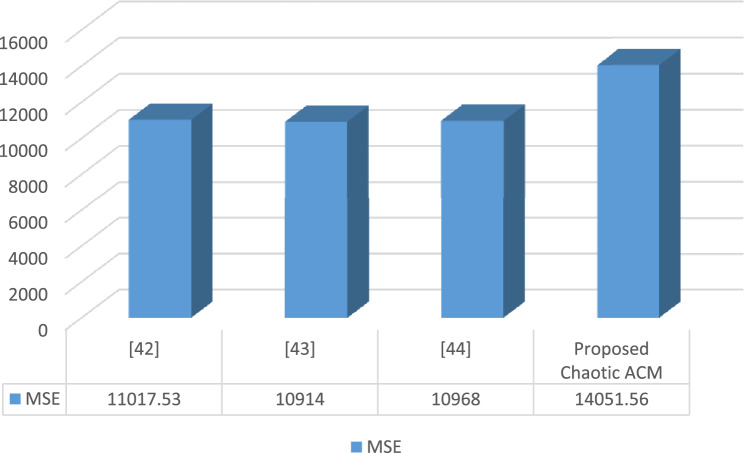
MSE analysis.

The accuracy and variance between two images are assessed using the mean square error (MSE). A high MSE score indicates significant variation between the original and encrypted images^31,40,41^. The MSE values are calculated using Eq. (16).

16 $$MSE=\frac{1}{{I}_{d}}\sum_{k=1}^{P}\sum_{l=1}^{Q}{(M\left(i,j\right)-N\left(i,j\right))}^{2}$$

where ${I}_{d}$ represents the dimension of the image, $M\left(i,j\right)$ represents the $(M\left(i,j\right))$ denotes the original medical image, and $(N\left(i,j\right))$ denotes the encoded image. Figure 7 shows the relationship among the MSE values of our proposed cryptosystem with some existing techniques like HNN-IES, BCE, Chaotic-Deep GAN.

#### Peak signal noise ratio (PSNR)

To compare the ciphered picture’s quality to the plain image, PSNR analysis is performed. A low PSNR value parallels to a significant change between the encrypted and the original image. PSNR can be investigated using Eq. (17).

17 $$PSNR=10.log\frac{{255}^{2}}{MSE}$$

#### Structural similarity index measure (SSIM)

**Figure 8:**
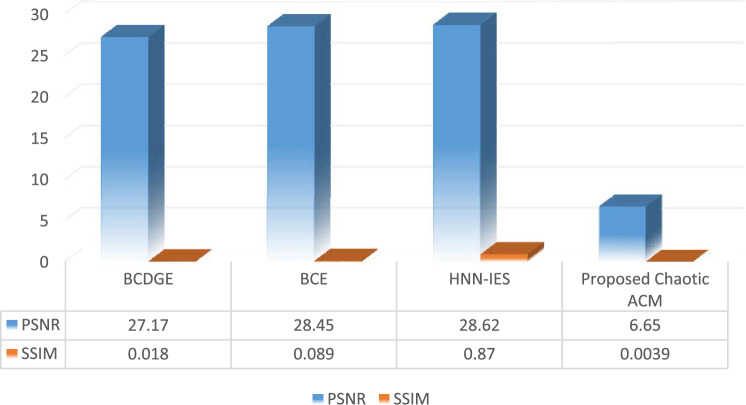
PSNR and SSIM analysis.

It is used to determine the resemblance among colored plain image and cipher image. Equation (18) is used to calculate the SSIM value for images.

18 $$SSIM=\frac{(2{\mu }_{{p}_{1}}{\mu }_{{p}_{2}}+{s}_{1})(2\delta {p}_{1}{p}_{2}+{s}_{2})}{({{\mu }_{{p}_{1}}}^{2}{{\mu }_{{p}_{2}}}^{2}+{s}_{1})({{\delta }_{{p}_{1}}}^{2}{{\delta }_{{p}_{2}}}^{2}+{s}_{2})}$$

where ${p}_{1}$ and ${p}_{2}$ indicates two images, $2\delta {p}_{1}{p}_{2}$ denotes the covariance of ${p}_{1}$ and ${p}_{2}$, ${{\delta }_{{p}_{1}}}^{2}$ denoted the variance of ${p}_{1}$, ${{\delta }_{{p}_{2}}}^{2}$ denotes the variance of ${p}_{2}$, ${\mu }_{{p}_{1}}$ denotes the mean value of ${p}_{1}$,${\mu }_{{p}_{2}}$ denotes the mean value of ${p}_{2}$, and ${s}_{1}$ and ${s}_{2}$ are constants to ensure stability of images. A greater SSIM score, which ranges from 0 to 1, denotes a high degree of similarity between two images. Our proposed BCAES is evaluated using 5 different medical images and comparison between values of Average PSNR and SSIM with some other schemes are represented in Fig. 8.

#### Time complexity

**Figure 9:**
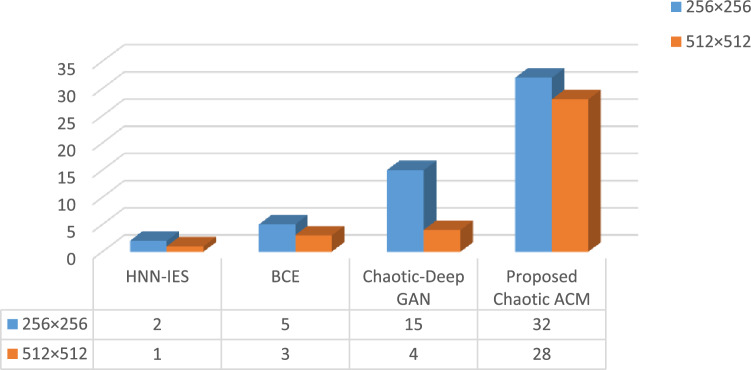
Analysis of time complexity.

The runtime of chaotic ACM encryption on medical photos of various resolutions is evaluated in Fig. 9 to evaluate the performance of the proposed network. At $256 \; \times \; 256$ resolution, our proposed encryption scheme can encrypt and decrypt 32 medical images per second, but 28 at $512\; \times \; 512$ resolution. On pictures of $512\; \times \;512$ and $256\; \times \;256$ resolution, our method has been demonstrated to give the shortest encryption times when compared to competing approaches like HNN-IES, BCE and BCDGE.

#### Differential attack analysis

Differential attacks are a kind of attacks where a criminal makes an effort to decrypt a picture without using private keys. For this, the intruder/attacker arbitrarily chooses a set of ordinary images, gain access to the encryption device to produce the corresponding encrypted images and then relates the cipher images to extract the mysterious data^42^. To ascertain the impact of one pixel value change in original image on the cipher image, a differential attack is employed. Therefore, the harder it is for attackers to figure out how to connect an encrypted image to a plain image, the greater the NPCR number. According to Fig. 6’s findings, the suggested encryption strategy has a greater NPCR value than the approaches currently in use, demonstrating its effectiveness in combating differential attacks.

#### Integrity and confidentiality analysis

A significant security problem is the privacy of healthcare information stored in the cloud. Cloud companies have taken measures to protect the secrecy of their data due to the high costs of reputation damage. Due to the availability of attackers, data secrecy in the cloud can’t be readily preserved and safeguarded. Analysis of the proposed system’s data integrity and confidentiality is therefore crucial. The suggested BCAES encrypts the medical images using three different private keys before sending them to the cloud server. Without these three different private keys, it is impossible to decrypt the encrypted image.

Moreover, the data saved on the cloud server can only be accessed by the organization or individual that has the data owner’s permission. As a result, only authorized individuals can decipher the ciphertext, protecting the privacy of the data^43,44^. Additionally, the signatures of each block guarantee data integrity. The blockchain network makes distinctions among different nodes and users based on their authenticity. The blockchain system ensures that only the authenticated user can decrypt the encrypted message using the secret keys by determining if the user has the right to do so.

## Conclusion

Cloud storage solutions are vulnerable to several security issues due to their openness. Careful analysis of the security measures is necessary when creating a cloud-based database for medical images. As a result, this study recommends BCAES, a secure architecture based on blockchain. Here, we suggested a revolutionary chaotic map-based image encryption method that will be saved in the cloud. The suggested method first generates a permutation phase using a Henon chaotic map. A Hill cypher with a key derived from an orthogonal matrix by considering a plane’s equation is employed for substitution. And then diffusion step uses an Arnold’s cat map (ACM) to create a sequence, which is bitwise XORed with each pixel’s value. The Henon map handles the confusion phase of the proposed algorithm’s operation, while the ACM handles the diffusion phase. The sender than signs the ciphertext’s ID puts it into the blockchain and uploads the encrypted image to the cloud. Later, the ciphertext image’s integrity may be confirmed using the signature. The suggested Chaotic ACM approach features three different keys, a large key space, and is particularly sensitive to alteration, according to experimental results and security studies. Comparing the BCAES architecture to other current methods, a high level of protection/security is thereby offered. Not only confidentiality but also authentication and integration of data. Table 6 shows different analysis results of our proposed scheme.

**Table 6:** Analysis results.

Analysis of image encryption scheme	BCE^28^	HNN-IES^25^	Chaotic deep GAN^20^	Proposed model
(a)				
Information Entropy	7.902	7.914	7.980	7.9992
NPCR	97.48	97.41	99.60	99.63
UACI	32.63	32.79	33.20	33.21
PSNR	28.45	28.62	27.117	6.65
SSIM	0.089	0.87	0.018	0.0039
Time complexity	5 images per second	2 images per second	15 images per second	32 images per second

Analysis of image encryption scheme	^30^	^39^	^42^	Proposed model
(b)				
Chi-square test	253.44	262.91	255.79	240.5

Analysis of image encryption scheme	^31^	^40^	^41^	Proposed model
(c)				
MSE	11,017.53	10,914	10,968	14,051.56

Though, use of classical chaotic systems have some inherent limitations, such as periodicity, easy destruction of phase space, and low lyapunov exponent. To handle these issues, many researchers have focused on improving classical chaotic systems to enhance their chaotic dynamic characteristics through a process called chaotification. The goal of chaotification is to make up for these limitations and improve the performance of chaotic encryption algorithms. As a future goal, this work may be modified or extended by replacing the classical chaotic maps with the maps obtained after the chaotification process.

## Data Availability

The data used to support the findings of this study are included within the article.
